# Editorial: preclinical data reproducibility for R&D - the challenge for neuroscience

**DOI:** 10.1186/2193-1801-4-1

**Published:** 2015-01-13

**Authors:** Thomas Steckler

**Affiliations:** Janssen Research & Development, Turnhoutseweg 30, 2340 Beerse, Belgium

The inability to reproduce published findings has been identified as a major issue in science. Reports of only a low percentage of landmark studies being reproduced at pharmaceutical companies like Bayer (Prinz et al. 2011) gained much interest in the scientific community and raised high levels of concerns. A more recent analysis from Amgen (Begley and Ellis 2012) suggested that those non-reproducible studies may have an even stronger impact on the field than those that can be reproduced, possibly because the more remarkable and exciting findings are reported in higher impact journals. Evidently, this is not just a problem of pharmaceutical industry. About half of respondents from faculty and trainees at the academic MD Anderson Cancer Center, Houston, Texas, had experienced at least one episode of inability to reproduce published data in a survey by Mobley et al. (2013) and comparable figures may be expected in neuroscience.

## Why worry?

Insufficient data reproducibility and integrity is a major concern, not only from a pure scientific perspective, but also because of potentially serious financial, legal and ethical consequences. It is currently estimated that up to 85% of resources are wasted in science (Chalmers and Glasziou 2009; Macleod et al. 2014). Investigational costs for a single case of misconduct may be in the range of US$ 525,000, amounting to annual costs exceeding US$ 100 MM for the US alone (Michalek et al. 2010). Such figures clearly contribute to a genuine dissatisfaction about the situation, also in the public domain, where questions on whether government spending on biomedical research is still justified are raised (The Economist 2013). In response, bodies like the Wellcome Trust or the Science Foundation Ireland implemented formal audit processes to combat misconduct and misuse of taxpayer’s money (Van Noorden 2014; Wellcome Trust 2013) and some research institutions where employees were directly involved in misconduct took drastic steps, including major re-organizations that affected large proportions of its staff (Normile 2014). Consequently, more transparency in reporting of preclinical data has been requested and best practices in experimental design and reporting proposed (Ioannidis 2014; Landis et al. 2012) - and in fact are urgently required!

The magnitude of the problem is further illustrated by a steep rise of retracted publications over the last years, with a high percentage suggested to be due to misconduct (fabrication and falsification, plagiarism or self-plagiarism) and more than 10% to be due to irreproducible data (Van Noorden 2011). The issue is not limited to published studies, although here the impact on the wider scientific community is possibly most severe. Problems were also observed in contract labs working for the pharmaceutical industry (Nature Medicine Opinions 2013; Selyukh and Yukhananov 2011) and industry itself is not without fault (e.g., Cyranoski 2013). The potential consequences for pharmaceutical industry are major and may lead from delays in drug development to potential retraction of drugs from the market, let alone the potential risks to human volunteers and patients.

This issue of reproducibility is highlighted against a background of increasing globalization of science and outsourcing activities from the pharmaceutical industry, with estimates that more than 30% of the annual business expenditure of pharma R&D in the US is spent on external research (Moris and Shackelford 2014) and projections that the global preclinical outsourcing market is still expanding, possibly more than doubling in growth from 2009 to 2016 (Mehta 2011). Whilst there are many advantages to externalize research, it also means people have to rely more on data generated by third parties that themselves may feel obliged to deliver what they think is expected by their customers. Furthermore, dealing with data from an external source adds an additional level of complexity to the already complex issue of data quality assurance. Conversely, in academia there is increasing pressure to deliver publications in order to be successful in the next grant acquisition (and as such future employment) or, one may argue, to be an interesting partner for industry.

## What are the issues at hand?

Partly driven by the situation of dwindling funding, many investigators are attracted to work in emerging and ‘hot’, but also very complex and competitive fields of science and like to use the most recent technology and innovative experimental designs. By taking this interesting approach, which may yield a lot of novel insights, there is a greater likelihood of receiving more favourable reviews of grant applications as well, especially as many grant schemes emphasize innovation rather than other aspects, such as reproducibility. Moreover, studies may get more rapidly published, often in so-called high impact journals, even if rather small and underpowered and, in this context, it may be more acceptable that reported effect sizes are small. However, all these factors diminish the positive predictive value of a study, i.e., the likelihood that results are true positives (Button et al. 2013; Ioannidis 2005). This issue is by no means limited to preclinical work or *in vivo* behavioural studies. It is also a concern for biomarker studies that play pivotal roles in drug discovery (Anderson and Kodukula 2014) and the many small explorative, clinical proof-of-concept studies often used to come to go/no-go decisions on drug development programs.

Often there is also an uncritical belief in p-values; over-reliance on highly significant, but also variable, p-values has been considered to be another important factor contributing to the high incidence of non-replication (Lazzeroni et al. 2014; Motulsky 2014; Nuzzo 2014). In general it is believed that expert statistical input is currently under-utilized and can help address issues of robustness and quality in preclinical research (Peers et al. 2012; 2014).

This ‘publish or perish’ pressure may also lead investigators to neglect findings, not conform to their hypothesis and instead to go for the desired outcome, may bias authors to publish positive, statistically significant results (Tsilidis et al. 2013) and to abandon negative results that they believe journals are unlikely to publish (the file-drawer phenomenon; Franco et al. 2014). This pressure to publish may even entice investigators to make *post-hoc* alterations to hypotheses, data, or statistics (Motulsky 2014; O’Boyle et al. 2014), so that there is a more compelling story to tell, essentially transforming uninteresting results into top-notch science (the chrysalis effect; O’Boyle et al. 2014). Reviewers of these manuscripts are also not free of bias, being possibly more willing to accept data that conform to their own scientific concepts; editors have an appetite for positive and novel findings rather than negative or ‘incremental’ results, and journals compete to publish breakthrough findings to boost their impact factor, which is calculated within the first two years of publication, whereas the n-year impact factor and the citation half-life receive considerably less attention. All of this, paired with the ease of publication in a world of electronic submissions and re-submissions with short turnaround times, generates a self-fulfilling, vicious circle. Unfortunately, there is no greater widely accepted forum where replication studies or negative studies can be published, although those data inevitably exist and are of equal importance to the field, let alone the ethical principles concerning repeated use of animals to show something does not work because publication of negative findings is discouraged.

Attempts to reproduce published findings are further hampered as many publications simply lack the detailed information required to reproduce experiments (Kilkenny et al. 2009). Indeed a recent analysis concluded that less than half of the neuroscience publications included in that analysis reported sufficient methodological detail to unanimously identify all materials/resources (Vasilevsky et al. 2013). Detailed information, however, is essential, especially in areas where tests and assays are not standardized and where there is high variability in experimental design and methodological detail across studies. This is frequently evident across many *in vivo* pharmacological reports (e.g., using different strains of rats or mice, sources of animals, housing conditions, size and made of test apparatus, habituation and training procedures, vehicles for drugs; e.g., Wahlsten 2001; Wahlsten et al. 2003), but *in vitro* studies may not fare much better either. Consequently, journals publishing original work must adhere to a minimum set of standards to even allow replication studies to be conducted, and many journals and editors have taken action to improve the information content provided in publications (McNutt 2014; Nature Editorial 2014), for example, by providing checklists that prompt authors to disclose important methodological details (Nature Editorial 2013).

The inability to reproduce due to lack of detailed information would possibly be less of an issue if data were robust. A robust finding should be detectable under a variety of experimental conditions, making obsolete the requirement for exact, point-by-point reproduction. It could possibly even be argued that most replication studies are in fact studies testing the robustness of reported findings, since it may be difficult to exactly recapitulate all details and conditions under which the original data were produced. Moreover, robust data could be considered more important as they can be seen under varying conditions and may be biologically more relevant. On the other hand, claims of non-reproducibility which do not utilise information that is provided in the original publication should also be carefully scrutinized to test the validity of the ‘replication’, which is often not the case. This in turn implies that we should not only encourage publication of reproduction attempts but also allow publications investigating the robustness of a reported effect and the validity of attempted replications.

While replication studies are usually performed by independent labs, replication attempts can of course also take place within the same laboratory, assessing the degree to which a test or assay produces stable and consistent results across experiments (intra-lab reliability). If intra-lab reliability is already low it comes as no surprise that reproducibility across labs (inter-lab reliability) is low as well, if not worse. Therefore, not only inter-lab replication studies, but also reports of attempts to systematically evaluate the intra-lab reliability of a particular test provide important information and publication of such data should be encouraged.

Particularly impacting the media, especially via the social media, are cases of fraud. Fraud or suspected fraud has been suggested to account for more than 40% of retracted papers in the biomedical sciences and life sciences (Fang et al. 2012), which is extremely alarming, although it is important to be reminded that the number of retracted articles is low compared to the huge number of articles that get published each year. However, a meta-analysis and systematic review of survey data concluded that close to 2% of scientists admitted to have fabricated, falsified or modified data or results at least once (Fanelli 2009). But contrary to fraudulent articles that are retracted upon detection of the misconduct, non-reproducible results hardly ever get retracted and yet may influence the field for years.

## What are the implications for neuroscience?

Because scientific advance is iterative, non-reproducibility, low reliability, lack of robustness and false discoveries have major implications, which go well beyond the waste of the taxpayer’s money. Researchers may waste their time and efforts, being misled by wrong assumptions, and that way may even jeopardize their future careers, but even more important is the loss of time for patients waiting for new therapies. Misguided research may lead to misdiagnosis, mistreatment and ill-advised development of new therapeutic approaches that lack efficacy and/or suffer from unacceptable side effects.

If negative data and failures to reproduce published work remain unshared, it essentially means that very valuable information for the field is withheld, potentially resulting in duplication of efforts, from which ethical questions arise, since in principle it contradicts one of the goals of the 3R’s (i.e., reduction) in animal research. Moreover, preclinical efficacy data are increasingly considered unreliable and being of low quality, especially behavioural data which, in many cases mistakenly, are considered nice-to-have rather than obligatory. Given the already very complex nature of neuroscientific research, with high demand for more effective therapies, coupled to low success rates to develop such therapies and high development costs (Frantz 2004; Kola and Landis 2004), there is disappointment in the lack of predictability and reliability of those data. As such there is an unwillingness to invest further in these areas and it may be speculated that this situation contributed, at least in part, to decisions of major pharmaceutical companies to exit the neuroscience field.

## Can we resolve the situation?

Recognizing this situation, a number of organizations have started to take action, including pharmaceutical companies, academia, governmental bodies, charities, editors and publishers (e.g., Landis et al. 2012; McNutt 2014; Nature Editorial 2014) and some scientists even took the initiative to replicate studies of critical data by independent labs prior to publication (Schooler 2014).

These are important steps towards improved data reproducibility. However, it is also very relevant to share the outcome of those activities more widely amongst scientists. While there are more instances now where efforts to reproduce published data can be shared with the scientific community (cf. some recent attempts to reproduce some findings reported with the drug bexarotene; Fitz et al. 2013; Price et al. 2013; Tesseur et al. 2013), those publications are still more an exception than the norm, yet provide very valuable information to the field. Fortunately, this is increasingly recognized and a number of programs have recently been launched to make it easier to publish studies aiming at reproducibility. One of these initiatives is a new Springer platform, focusing on publications of peer-reviewed studies concerned with reproduction of recently reported findings in the neuroscience area. This section, which is called “Replication Studies in Neuroscience”, is part of the open access, electronic SpringerPlus journal (http://www.springerplus.com/about/update/RepStudNeuro). Neuroscientists, including the readers of Psychopharmacology, should feel encouraged to submit replication studies to journals like this. Sharing these results is highly relevant to Psychopharmacology, both to the research field and to the journal, as it hopefully will help to increase the positive predictive value of our tests and assays, will contribute to scientific quality and eventually help to re-build trust in research and neuroscience in general.

Although this article makes a plea for greater emphasis on reproducibility, there should also not be a shift to an aggressively sceptical tendency where some scientists make their names by failing to repeat others’ work or where careers of brilliant young scientists are jeopardized because someone else published an article failing to reproduce a particular result. This can be a very intimidatory and threatening situation for many excellent scientists working in good faith to produce robust and useful data. The quest for reproducibility needs to be conducted in a scientific and ethical manner which pays careful attention to its consequences. But what is needed is a cultural change that puts more emphasis on the value of data reproducibility, reliability and robustness of data, rather than just novelty aspects. We hope initiatives like the ones mentioned above can make a contribution to this endeavour.
